# Effects of intracranial atherosclerosis and atrial fibrillation on the prognosis of ischemic stroke with active cancer

**DOI:** 10.1371/journal.pone.0259627

**Published:** 2021-11-05

**Authors:** Ki-Woong Nam, Hyung-Min Kwon, Yong-Seok Lee

**Affiliations:** 1 Department of Neurology, Seoul Metropolitan Government-Seoul National University Boramae Medical Center, Seoul, South Korea; 2 Department of Neurology, Seoul National University College of Medicine, Seoul, South Korea; Universita degli Studi di Roma La Sapienza, ITALY

## Abstract

**Background:**

In ischemic stroke patients with active cancer, cryptogenic stroke has worse prognosis than stroke by conventional mechanisms. However, the individual effects of intracranial atherosclerosis (ICAS) or atrial fibrillation (AF) on the prognosis of these patients have not been studied.

**Aims:**

Therefore, we aimed to investigate the effects of ICAS and AF on the prognosis of ischemic stroke patients with active cancer.

**Methods:**

We included ischemic stroke patients with active cancer between 2010 and 2020. Early neurological deterioration (END) was defined as an increase of ≥ 1 in the motor NIHSS score, or ≥ 2 in the total NIHSS score within 72 hours of admission. Unfavorable outcomes were defined as a score of ≥ 3 on the 3-month modified Rankin Scale.

**Results:**

In total, 116 ischemic stroke patients with active cancer were evaluated. In multivariable analysis, ICAS was positively associated with END (adjusted odds ratio [aOR] = 4.56, 95% confidence interval [CI]: 1.52–13.70), and this association showed a quantitative relationship according to the degree of stenosis of ICAS (stenosis group: aOR = 4.24, 95% CI: 1.31–13.72; occlusion group, aOR = 5.74, 95% CI: 1.05–31.30). ICAS was also closely related to unfavorable outcomes (aOR = 6.33, 95% CI: 1.15–34.79). In contrast, AF showed no significant association with END or unfavorable outcomes. Our data showed that patients with ICAS had larger and more severe initial stroke lesions, and poorer prognosis than those without.

**Conclusions:**

ICAS, but not AF, was closely associated with poor prognosis in ischemic stroke patients with active cancer.

## Introduction

Cancer and ischemic stroke are the leading causes of disability and death worldwide [1, 2]. Both these two diseases are closely related, and active cancer has now been recognized as an independent risk factor for ischemic stroke [1–3]. Ischemic stroke is common in cancer patients, and up to 15% of them experience it in their clinical course [1, 4]. Furthermore, ischemic stroke in cancer patients has complicated causes of occurrence because both cancer-specific and conventional stroke mechanisms are involved [1, 4, 5]. Therefore, strokes that occur in them are more severe, recur more frequently, and have a worse prognosis than in other patients [4, 6–8].

As the life expectancy of cancer patients has increased dramatically, their long-term quality of life has become an important issue [4, 9]. Thus, ischemic stroke in cancer patients, which can cause permanent disability or death, has been studied actively. In the results of previous studies, the characteristics of ischemic stroke with active cancer were evident in patients with cryptogenic stroke of the Trial of ORG 10172 in Acute Stroke Treatment (TOAST) classification or in those with fewer conventional vascular risk factors [5, 10]. However, these studies compared the prognosis by simply dividing the patients into a cryptogenic mechanism group and a conventional mechanism group, and the latter additionally included patients with small-vessel occlusion with a good prognosis [5, 10, 11]. Therefore, the actual effects of intracranial atherosclerosis (ICAS) or atrial fibrillation (AF) on the prognosis of ischemic stroke in patients with active cancer may have been masked.

Generally, ischemic stroke caused by ICAS has poor prognosis [12, 13]. Additionally, the brain environment created by ICAS is sufficient to produce negative synergy with cancer-specific stroke mechanisms, such as intravascular coagulopathy or nonbacterial thrombotic endocarditis (NBTE) [1, 9]. In the case of AF, it can provide opportunities for additional embolism along with the thromboembolism produced by cancer [14]. Moreover, ICAS, AF and cancer share risk factors, and ICAS and AF are frequently observed in patients with advanced cancer [4]. Therefore, our hypothesis that, in cancer patients, ischemic stroke with ICAS or AF will have a worse prognosis than those with stroke by cryptogenic mechanisms seems worth testing. In this study, we examined the association of ICAS and AF with the prognosis of ischemic stroke in patients with active cancer.

## Material and methods

### Study population

From a consecutive stroke registry of a large medical center in Korea (Seoul Metropolitan Government-Seoul National University Boramae Medical Center), we included ischemic stroke patients with active cancer within 7 days of symptom onset between January 2010 and December 2020. As in previous studies, active cancer was defined when there was a new diagnosis of, recurrence or progression of, or treatment for cancer within 6 months prior to enrollment [5, 6, 15]. To examine the clinical implications of ICAS and AF in ischemic stroke patients with active cancer, we excluded patients with the following stroke mechanisms based on the TOAST classification: 1) extracranial large artery atherosclerosis (with occlusion or ≥ 50% stenosis of the relevant vessel); 2) small vessel occlusion; and 3) other determined etiology [16]. The additional exclusion criteria were age < 18 years, hematologic or primary brain cancer with stroke mechanisms different from those of solid cancer, and brain magnetic resonance imaging (MRI) not performed [5–7]. In total, 116 patients with ischemic stroke were included in the final analysis.

This study was approved by the Institutional Review Board of the Seoul Metropolitan Government-Seoul National University Boramae Medical Center (number: 20-2021-35). The requirement to obtain informed consent from participants was waived by the Institutional Review Board because of the retrospective study design using anonymous and de-identified information. All experiments were performed in accordance with the Declaration of Helsinki and relevant guidelines and regulations. All data and materials related to this article are included in the main text and supplemental materials.

### Demographic, clinical, and laboratory assessments

We evaluated the demographic and clinical characteristics of patients, including age, sex, hypertension, diabetes, dyslipidemia, AF, current smoking, initial stroke severity, and thrombolytic therapy. AF was documented by electrocardiogram, 24-hour Holter monitoring, or continuous electrocardiogram monitoring during admission. The initial stroke severity was rated using the National Institutes of Health Stroke Scale (NIHSS) on a daily basis by appropriately trained neurologists who were not involved in this study. Cancer-related information such as types of cancer, systemic metastasis, and adenocarcinoma was also evaluated [6].

This study evaluated the acute to subacute prognosis of ischemic stroke patients with active cancer based on the early neurological deterioration (END) and 3-month modified Rankin Scale (3m-mRS) scores. The END was defined as an increase of ≥ 1 in the motor NIHSS score or ≥ 2 in the total NIHSS score within the first 72 hours of admission [6]. Unfavorable outcome was defined as a score of ≥ 3 on the 3m-mRS (3m-mRS: 3–6).

### Radiological assessments

All study population underwent brain MRI and magnetic resonance angiography (MRA) within 24 hours of admission using a 1.5-T MR scanner (Achieva 1.5 T; Philips, Eindhoven, Netherlands). The detailed MRI acquisitions were as follows: diffusion-weighted imaging (DWI) [repetition time (TR)/echo time (TE) = 3,000/44 ms], T1-weighted images (TR/TE = 500/11 ms), T2-weighted images (TR/TE = 3,000/100 ms), T2-gradient echo images (TR/TE = 57/20 ms), fluid-attenuated inversion recovery images (TR/TE = 3,000/100 ms), and three-dimensional time-of-flight MRA (TR/TE = 24/3.5 ms, slice thickness = 1.2 mm). The basic slice thickness, except for the time-of-flight MRA was 5 mm. We classified the patterns of initial DWI lesions into single and multiple territory lesions [6, 17]. Moreover, we quantitatively measured the DWI lesion volumes using a computer-assisted semi-automated technique (MIPAV, version 7.3.0, National Institutes of Health, Bethesda, MD) [18]. ICAS was defined as occlusion or ≥ 50% stenosis of the intracranial vessels on the time-of-flight MRA images [19]. We classified the ICAS lesion burden as absent, stenosis, or occlusion according to the degree of stenosis [20]. To distinguish between ICAS and acute occlusion by thrombus, we referred to previous MRA images, checked for the presence of any signs of clots on the T2-gradient echo images, or recanalization in the follow-up MRA images, and referred to conventional angiography data. All radiological assessments were rated by two well-trained neurologists (K.-W.N. and Y.-S.L.), and disagreements were resolved by discussion with a third rater (H.-M.K.).

### Statistical analysis

All statistical analyses were performed using SPSS version 20.0 (IBM, SPSS, Armonk, NY, USA). Univariate analyses were performed to identify the possible predictors of END. Considering the definition of END, we performed an analysis on 107 patients in the study population who visited the hospital within 72 hours after symptom onset. For these analyses, the Student’s *t*-test or the Mann-Whitney *U*-test was used for continuous variables and the Chi-squared test or Fisher’s exact test for categorical variables. Based on the results of the univariate analyses, variables with *P* < 0.10 were introduced into the multivariable logistic regression analysis as confounders. Continuous variables with skewed data were transformed using a log scale. The DWI lesion volume and thrombolytic therapy were not included in the multivariable analysis because of the severe correlation with the initial NIHSS score. Of the total 116 study population, 6 patients did not have 3m-mRS score data. Therefore, in order to find possible predictors of unfavorable outcomes, we analyzed 110 participants in the same way as END.

To compare the quantitative relationship between the ICAS lesion burden and the prognosis, we compared the characteristics of the patients according to the degree of stenosis of ICAS. Furthermore, we compared the characteristics and prognosis among the following four patient groups to help interpret the objective of this study: [cryptogenic], [only AF], [only ICAS], and [both AF and ICAS] groups. To perform these analyzes, the chi-squared test was used for categorical variables. For continuous variables, the Kruskal-Wallis test for statistical differences and the Jonckheere-Terpstra test for statistical trends were used together. All variables with *P* < 0.05 were considered statistically significant.

## Results

In total, 116 ischemic stroke patients with active cancer were evaluated (mean age, 71 ± 11 years; males, 61.2%; and mean initial NIHSS score, 9 ± 7). The END events occurred in 32 (27.6%) patients, and 50 (43.1%) presented unfavorable outcomes. AF was detected in 21 (18.1%) patients of the study population, while ICAS was detected in 37 (31.9%), of whom 26 (22.4%) had stenosis and 11 (9.5%) had occlusion. Other detailed baseline characteristics are shown in S1 Table.

In univariate analysis, END showed a statistically significant correlation with initial NIHSS score, thrombolytic therapy, and ICAS, and a statistically close tendency with AF, high-sensitivity C-reactive protein (hs-CRP), MRI lesion pattern, and DWI lesion volume (Table 1). In multivariable logistic regression analysis, ICAS (adjusted odds ratio [aOR] = 4.56, 95% confidence interval [CI]: 1.52–13.70) and multiple territory lesion (aOR = 2.90, 95% CI: 1.05–7.97) remained significant after adjusting for confounders. ICAS also showed a quantitative positive correlation with END according to the degree of stenosis (stenosis group: aOR = 4.24, 95% CI: 1.31–13.72; occlusion group: aOR = 5.74, 95% CI: 1.05–31.30). AF showed a positive association with END, but it was not statistically significant (aOR = 3.31, 95% CI: 0.97–11.32; Table 2).

**Table 1:** Baseline characteristics of patients with and without early neurological deterioration (n = 107).

	No END	END	*P* value
Number	76	31	
Age, y [SD]	71 ± 11	72 ± 11	0.712
Visit time, h [IQR]	4.8 [1.5–22.3]	4.0 [1.0–10.0]	0.172
Sex, male, n (%)	47 (61.8)	18 (58.1)	0.717
Hypertension, n (%)	54 (71.1)	18 (58.1)	0.194
Diabetes, n (%)	24 (31.6)	14 (45.2)	0.183
Dyslipidemia, n (%)	23 (30.3)	14 (45.2)	0.142
Atrial fibrillation, n (%)	11 (14.5)	9 (29.0)	0.080
Current smoking, n (%)	19 (25.0)	8 (25.8)	0.931
Cancer type, n (%)			0.566
Lung	24 (31.6)	7 (22.6)	
Gastric/esophageal	10 (13.2)	4 (12.9)	
Colorectal	6 (7.9)	5 (16.1)	
Hepatobiliary	17 (22.4)	5 (16.1)	
Genitourinary	10 (13.2)	4 (12.9)	
Breast	4 (5.3)	1 (3.2)	
Others	5 (6.6)	5 (16.1)	
Systemic metastasis, n (%)	43 (56.6)	16 (51.6)	0.639
Adenocarcinoma, n (%)	34 (55.7)	12 (54.5)	0.923
Systolic BP, mmHg [SD]	142 ± 23	139 ± 24	0.490
Diastolic BP, mmHg [SD]	81 ± 12	80 ± 12	0.598
Initial NIHSS score [IQR]	6 [2–12]	14 [5–18]	0.006
Thrombolytic therapy, n (%)	5 (6.6)	6 (19.4)	0.048
White blood cell, x 10^3^/μL [IQR]	7.12 [5.32–10.23]	7.89 [6.14–12.07]	0.112
High sensitivity CRP, mg/dL [IQR]	0.63 [0.17–2.59]	1.93 [0.36–7.56]	0.076
D-dimer, μg/mL [IQR]	2.37 [1.08–8.90]	3.74 [0.82–8.93]	0.642
MRI lesion pattern, n (%)			0.087
Single territory	48 (63.2)	14 (45.2)	
Multiple territory	28 (36.8)	17 (54.8)	
DWI lesion volume, mL [IQR]	5.17 [0.83–24.79]	13.35 [3.75–40.01]	0.059
Intracranial atherosclerosis, n (%)	17 (22.4)	17 (54.8)	0.001

END = early neurological deterioration, BP = blood pressure, NIHSS = National Institutes of Health Stroke Scale, CRP = C-reactive protein, MRI = magnetic resonance imaging, DWI = diffusion-weighted imaging.

**Table 2:** Multivariable logistic regression for possible predictors of early neurological deterioration.

	Crude OR (95% CI)	*P*-value	Adjusted OR (95% CI)	*P*-value
Model 1				
Atrial fibrillation	2.42 [0.89–6.60]	0.085	3.31 [0.97–11.32]	0.056
Initial NIHSS score	1.09 [1.03–1.15]	0.005	1.02 [0.95–1.10]	0.596
High sensitivity CRP*	1.25 [1.00–1.56]	0.055	1.10 [0.84–1.45]	0.497
Multiple territory lesion	2.08 [0.89–4.86]	0.090	2.90 [1.05–7.97]	0.039
Intracranial atherosclerosis	4.21 [1.73–10.26]	0.002	4.56 [1.52–13.70]	0.007
Model 2				
Atrial fibrillation	2.42 [0.89–6.60]	0.085	3.26 [0.95–11.22]	0.060
Initial NIHSS score	1.09 [1.03–1.15]	0.005	1.02 [0.94–1.10]	0.697
High sensitivity CRP*	1.25 [1.00–1.56]	0.055	1.11 [0.84–1.46]	0.478
Multiple territory lesion	2.08 [0.89–4.86]	0.090	2.87 [1.04–7.91]	0.042
Intracranial atherosclerosis		0.004		0.025
Absent	Ref	Ref	Ref	Ref
Stenosis	3.24 [1.18–8.90]	0.022	4.24 [1.31–13.72]	0.016
Occlusion	7.38 [1.89–28.72]	0.004	5.74 [1.05–31.30]	0.043

NIHSS = National Institutes of Health Stroke Scale, CRP = C-reactive protein.

*This variable was transformed using a log scale.

The unfavorable outcome showed a statistically significant association with systemic metastasis, initial NIHSS score, thrombolytic therapy, white blood cell (WBC) counts, hs-CRP, D-dimer, MRI lesion pattern, DWI lesion volume, and ICAS and a close tendency with diabetes and AF (Table 3). In multivariable analysis, ICAS (aOR = 6.33, 95% CI: 1.15–34.79) remained significant after adjusting for confounders. The initial NIHSS score (aOR = 1.22, 95% CI: 1.09–1.37) and multiple territory lesion (aOR = 19.31, 95% CI: 3.61–103.32) were also significantly associated with unfavorable outcomes, independent of ICAS. AF did not show a statistically significant association with unfavorable outcomes (Table 4).

**Table 3:** Comparisons between patients with favorable and unfavorable outcomes (n = 110).

	Favorable (3m-mRS 0–2)	Unfavorable (3m-mRS 3–6)	*P* value
Number	60	50	
Age, y [IQR]	72 [62–79]	73 [66–79]	0.599
Visit time, h [IQR]	6.0 [2.0–27.5]	4.3 [1.0–20.0]	0.149
Sex, male, n (%)	38 (63.3)	30 (60.0)	0.720
Hypertension, n (%)	43 (71.7)	31 (62.0)	0.282
Diabetes, n (%)	18 (30.0)	23 (46.0)	0.084
Dyslipidemia, n (%)	20 (33.3)	19 (38.0)	0.610
Atrial fibrillation, n (%)	8 (13.3)	13 (26.0)	0.092
Current smoking, n (%)	17 (28.3)	11 (22.0)	0.448
Cancer type, n (%)			0.358
Lung	19 (31.7)	14 (28.0)	
Gastric/esophageal	8 (13.3)	5 (10.0)	
Colorectal	5 (8.3)	6 (12.0)	
Hepatobiliary	8 (13.3)	15 (30.0)	
Genitourinary	10 (16.7)	6 (12.0)	
Breast	4 (6.7)	1 (2.0)	
Others	6 (10.0)	3 (6.0)	
Systemic metastasis, n (%)	25 (41.7)	32 (64.0)	0.020
Adenocarcinoma, n (%)	27 (52.9)	23 (63.9)	0.309
Systolic BP, mmHg [SD]	143 ± 24	138 ± 23	0.243
Diastolic BP, mmHg [SD]	82 ± 12	80 ± 12	0.434
Initial NIHSS score [IQR]	3 [1–6]	14 [6–19]	< 0.001
Thrombolytic therapy, n (%)	1 (1.7)	10 (20.0)	0.001
HbA1c, % [IQR]	5.8 [5.4–6.3]	6.0 [5.7–6.6]	0.115
Total cholesterol, mg/dL [SD]	153 ± 35	159 ± 58	0.547
White blood cell, x 10^3^/μL [IQR]	6.04 [5.00–8.48]	8.79 [6.30–12.52]	< 0.001
hs-CRP, mg/dL [IQR]	0.42 [0.10–1.46]	2.32 [0.41–7.56]	< 0.001
D-dimer, μg/mL [IQR]	1.82 [0.60–3.83]	4.63 [1.96–13.34]	< 0.001
MRI lesion pattern, n (%)			< 0.001
Single territory	46 (76.7)	19 (38.0)	
Multiple territory	14 (23.3)	31 (62.0)	
DWI lesion volume, mL [IQR]	1.85 [0.39–10.21]	11.96 [4.20–56.51]	< 0.001
Intracranial atherosclerosis, n (%)	11 (18.3)	24 (48.0)	0.001

3m-mRS = 3-month modified Rankin Scale, BP = blood pressure, NIHSS = National Institutes of Health Stroke Scale, HbA1c = hemoglobin A1c, hs-CRP = high sensitivity c-reactive protein, MRI = magnetic resonance imaging.

**Table 4:** Multivariable logistic regression for possible predictor of unfavorable outcomes (3-month modified Rankin Scale score, 3–6).

	Crude OR (95% CI)	*P*-value	Adjusted OR (95% CI)	*P*-value
Diabetes	1.99 [0.91–4.35]	0.086	1.09 [0.28–4.30]	0.905
Atrial fibrillation	2.28 [0.86–6.06]	0.097	2.17 [0.37–12.85]	0.392
Systemic metastasis	2.49 [1.15–5.39]	0.021	0.69 [0.17–2.87]	0.614
Initial NIHSS score	1.25 [1.15–1.36]	< 0.001	1.22 [1.09–1.37]	0.001
High sensitivity CRP*	1.60 [1.26–2.02]	< 0.001	1.29 [0.86–1.92]	0.219
D-dimer*	1.76 [1.26–2.45]	0.001	1.03 [0.59–1.81]	0.916
Multiple territory lesion	5.36 [2.35–12.26]	< 0.001	19.31 [3.61–103.32]	0.001
Intracranial atherosclerosis	4.11 [1.74–9.69]	0.001	6.33 [1.15–34.79]	0.034

mRS = modified Rankin Scale, NIHSS = National Institutes of Health Stroke Scale, CRP = C-reactive protein.

*These variables were transformed using a log scale.

**Fig 1:**
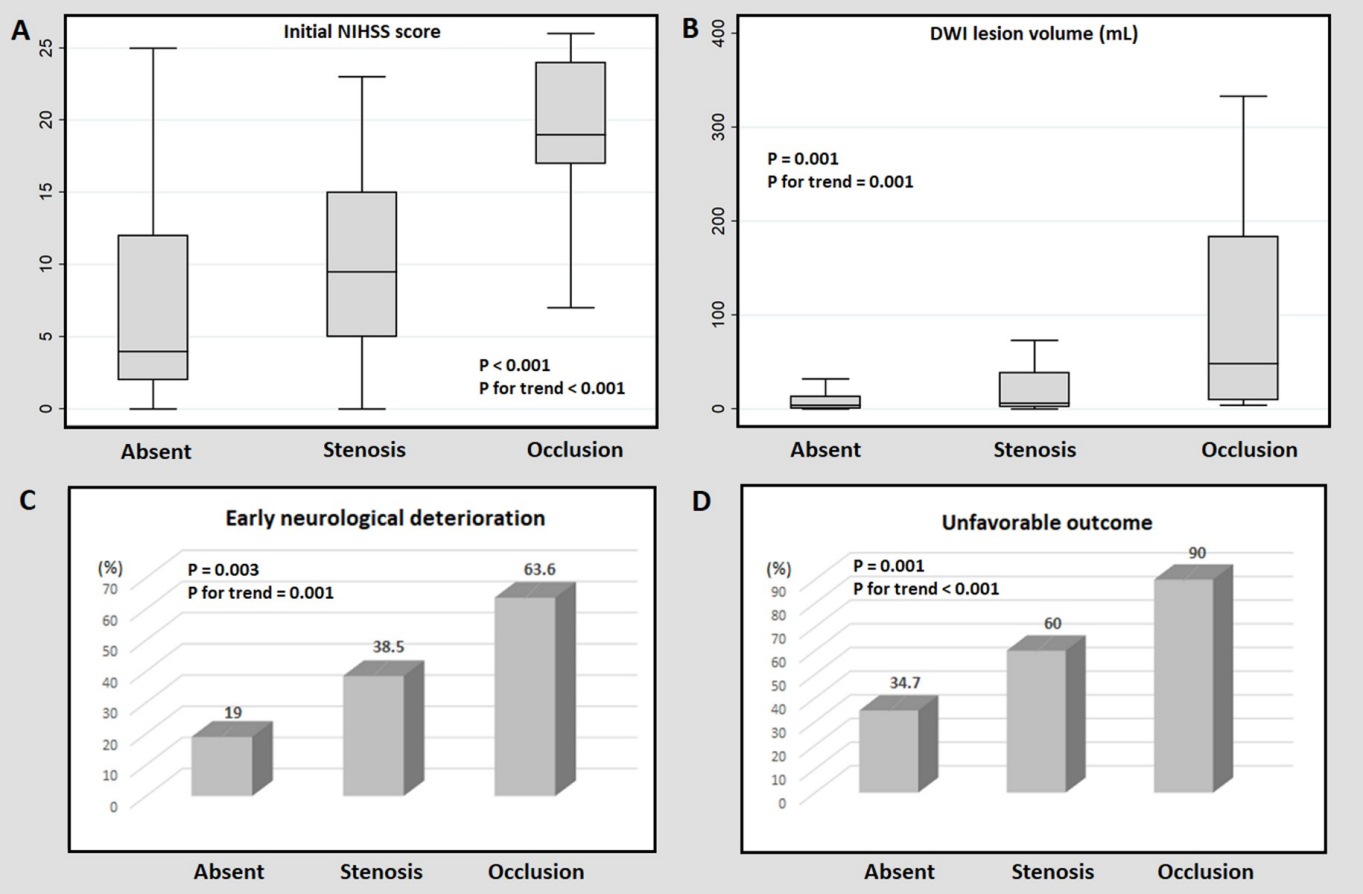
Comparison of the prognosis according to the intracranial atherosclerosis lesion burden. Patients with occlusive ICAS lesions had higher initial NIHSS scores (A), larger DWI lesion volumes (B), more frequent END, (C) and more unfavorable outcomes (D) as compared to the other patients. ICAS = intracranial atherosclerosis, NIHSS = National Institutes of Health Stroke Scale, DWI = diffusion-weighted imaging, END = early neurological deterioration.

When comparing the prognosis according to the ICAS lesion burden, the stenosis degree positively correlated with the initial NIHSS score (*P* for trend < 0.001), DWI lesion volume (*P* for trend = 0.001), END (*P* for trend = 0.001), and unfavorable outcomes (*P* for trend < 0.001) (Fig 1).

Furthermore, we examined the individual and synergistic influences on the prognosis of ICAS and AF by comparing the characteristics of the four groups. The group with [both AF and ICAS] showed higher frequencies of END and unfavorable outcomes and higher initial NIHSS score, WBC counts, hs-CRP, and DWI lesion volume as compared to the other groups. The distribution of these trends in the descending order of the groups was [both AF and ICAS], [only ICAS], [only AF], and [cryptogenic] groups. The D-dimer level or multiple territory lesion did not seem to differ significantly among the four groups (Table 5).

**Table 5:** Comparisons of characteristics in ischemic stroke patients with active cancer according to the presence or absence of atrial fibrillation and intracranial atherosclerosis.

	Cryptogenic	Only AF	Only ICAS	Both AF and ICAS	P value	P for trend
Number	64	15	31	6		
END, n (%)	11 (17.2)	4 (26.7)	11 (35.5)	6 (100.0)	< 0.001	< 0.001
Unfavorable outcome, n (%)	19 (31.7)	7 (46.7)	18 (62.1)	6 (100.0)	0.002	< 0.001
Initial NIHSS, [IQR]	4 [2–11]	6 [3–14]	11 [5–18]	23 [16–25]	< 0.001	< 0.001
Age, y [IQR]	72 [62–79]	79 [72–84]	73 [61–78]	71 [61–81]	0.084	0.679
Sex, male, n (%)	38 (59.4)	8 (53.3)	21 (67.7)	4 (66.7)	0.773	0.463
Systemic metastasis, n (%)	34 (53.1)	7 (46.7)	20 (64.5)	1 (16.7)	0.167	0.885
WBC counts, x 10^3^/μL [IQR]	6.56 [5.32–9.98]	6.75 [6.03–8.94]	9.24 [5.92–13.10]	9.63 [6.28–12.74]	0.075	0.013
High sensitivity CRP, mg/dL [IQR]	0.54 [0.17–2.91]	0.91 [0.39–2.42]	2.23 [0.39–7.56]	3.09 [0.21–16.45]	0.192	0.037
D-dimer, μg/mL [IQR]	2.36 [0.79–13.01]	3.14 [1.99–4.70]	3.55 [1.98–8.90]	3.16 [0.40–3.92]	0.742	0.647
Multiple territory lesion, n (%)	32 (50.0)	5 (33.3)	10 (32.3)	2 (33.3)	0.317	0.086
DWI lesion volume, mL [IQR]	3.52 [0.52–11.96]	5.34 [1.74–30.71]	13.35 [3.31–48.33]	33.60 [8.49–187.31]	0.008	0.001

AF = atrial fibrillation, ICAS = intracranial atherosclerosis, END = early neurological deterioration, NIHSS = National Institutes of Health Stroke Scale, WBC = white blood cell, CRP = C-reactive protein, DWI = diffusion-weighted imaging.

## Discussion

In this study, we found that ICAS was associated with END and unfavorable outcomes in ischemic stroke patients with active cancer and that these associations were proportional to the degree of stenosis of ICAS. Moreover, large size and high severity index strokes were found in patients with severe ICAS lesions. In contrast, patients with AF seemed to have poor prognosis, but the difference was not statistically significant.

The exact mechanisms explaining the close relationship between ICAS and END or unfavorable outcomes are unclear. However, we suggested several plausible hypotheses. First, ICAS itself may act as a pathology leading to a poor prognosis of stroke. ICAS is already known to cause frequent early stroke recurrence and END in general stroke patients without cancer [21]. In addition, it induces a chronic perfusion defect, creating a brain environment vulnerable to ischemic insult, leading to larger and more severe strokes [22, 23]. In our data, as the severity of ICAS increased (i.e., absent group, stenosis group, and occlusion group), higher initial NIHSS scores and more frequent ENDs were found (Table 2 and Fig 1). This may mean the pathological action of ICAS is also working in ischemic stroke patients with active cancer. Second, if a cancer-specific stroke mechanism occurs in the area narrowed by ICAS, it may lead to a worse prognosis. In cancer patients, as mentioned earlier, thrombus may be formed by intravascular coagulopathy or embolism may occur due to NBTE [1, 9, 17]. When thrombus or emboli reach the site of ICAS, larger initial strokes, distal embolization through recanalization, and hemorrhagic transformation are likely to occur compared to patients without ICAS. Supporting this hypothesis, our data showed that patients with ICAS presented a distinctly larger DWI lesion volume and a higher tendency for post-stroke inflammation than those without ICAS (Table 5). The initial NIHSS score and DWI lesion volume increased in proportion to the degree of stenosis of the ICAS, and the prognosis was worse (Fig 1). In addition, frequent multiple territory lesions and systemic metastasis, and high d-dimer levels were observed in patients with END regardless of whether they had ICAS or not. These findings give the impression that the poor prognosis of our patients is a synergistic effect with cancer-specific thromboembolism rather than a single effect of ICAS. However, apparently, ICAS is also a potent poor prognostic factor, and our current results alone cannot conclude which of the two mechanisms is correct. To elucidate this, a further study looking at comparisons between ICAS patients with/without cancer is needed. Last, ICAS may be an indicator of more advanced cancer with a poor prognosis. It is already known that advanced cancer is associated with atherosclerosis. Also, in our data, although not statistically significant, patients with ICAS showed a higher frequency of systemic metastasis than those without. Therefore, ICAS can be thought of as an indicator found in patients originally with poor prognoses.

Participants with AF also had larger and more severe initial stroke lesions, more frequent ENDs, and worse prognoses than those with cryptogenic stroke. Since the thrombi produced in the heart due to AF are larger in size than the intravascular thrombi caused by active cancer [1, 17, 24], it is not unusual for these patients to develop large and serious initial stroke lesions. Furthermore, if active cancer patients who are already prone to thromboembolism have AF, there are additional opportunities for embolism and early progression is likely to occur [14]. However, in our study, AF showed a close tendency with END/unfavorable outcomes, but the difference was not statistically significant. Thus, the influence of AF appears to be less pronounced than that of ICAS.

### Limitations

There are several limitations in our study. First, since this study followed a retrospective cross-sectional design, we can only discuss associations and not the causality. Second, some patients with occlusive ICAS have the potential for acute occlusion. We used all available means to minimize this possibility, such as checking previous MRA findings and presence of clot signs and recanalization. It would have been more accurate if conventional angiography was performed for all patients, but this is not feasible in a retrospective study design. Third, if follow-up MRI results were available, it would have helped to explain the mechanism of how ICAS actually causes END. Fourth, the impact of non-neurological complications including stroke-associated pneumonia should be considered [25, 26]. Last, though we used a relatively sensitive definition of END [27], since ICAS showed a statistically significant association with unfavorable outcomes based on the 3-month mRS scores, we can interpret that it exerts sufficient influence on the prognosis.

## Conclusion

We demonstrated that ICAS was closely associated with poor prognosis during the acute to subacute period in ischemic stroke patients with active cancer. This association appeared to be related to the tendency to form large and severe initial stroke lesions when accompanied by ICAS. Therefore, by performing the initial MRA together, we can help classify high-risk groups with poor prognosis later. However, further prospective studies are needed to validate our results.

## Supporting information

S1 Table Baseline characteristics of the study population (n = 116). (DOCX)
