# Supplementary material for: Effects of intracranial atherosclerosis and atrial fibrillation on the prognosis of ischemic stroke with active cancer
Source: PLoS One. 2021 Nov 5;16(11):e0259627. doi: 10.1371/journal.pone.0259627 (PMC8570487; doi:10.1371/journal.pone.0259627)
Supplement: S1 Table — (DOCX) [file pone.0259627.s001.docx]

**S1 Table. Baseline characteristics of the study population (n = 116)**

| Age, y [SD] | 71 ± 11 |
| --- | --- |
| Visit time, h [SD] | 23.6 ± 39.9 |
| Sex, male, n (%) | 71 (61.2) |
| Hypertension, n (%) | 78 (67.2) |
| Diabetes, n (%) | 43 (37.1) |
| Dyslipidemia, n (%) | 41 (35.3) |
| Atrial fibrillation, n (%) | 21 (18.1) |
| Current smoking, n (%) | 29 (25.0) |
| Cancer type, n (%) |  |
| Lung | 33 (28.4) |
| Gastric/esophageal | 14 (12.1) |
| Colorectal | 12 (10.3) |
| Hepatobiliary | 26 (22.4) |
| Genitourinary | 16 (13.8) |
| Breast | 5 (4.3) |
| Others | 10 (8.6) |
| Systemic metastasis, n (%) | 62 (53.4) |
| Adenocarcinoma, n (%) | 52 (57.1) |
| Systolic BP, mmHg [SD] | 142 ± 24 |
| Diastolic BP, mmHg [SD] | 81 ± 12 |
| Initial NIHSS score [SD] | 9 ± 7 |
| Thrombolytic therapy, n (%) | 11 (9.5) |
| White blood cell, x 10^3^/µL [SD] | 8.41 ± 4.07 |
| High-sensitivity CRP, mg/dL [SD] | 4.11 ± 6.80 |
| D-dimer, µg/mL [SD] | 7.41 ± 10.22 |
| MRI lesion pattern, n (%) |  |
| Single territory | 67 (57.8) |
| Multiple territory | 49 (42.2) |
| DWI lesion volume, mL [SD] | 26.95 ± 51.06 |
| Intracranial atherosclerosis, n (%) | 37 (31.9) |
| Early neurological deterioration, n (%) | 32 (27.6) |
| 3-months Modified Rankin Scale [SD] | 3 ± 2 |

NIHSS = National Institutes of Health Stroke Scale, CRP = c-reactive protein, MRI = magnetic resonance imaging, WMH = white matter hyperintensity
